# Decyl caffeate inhibits the proliferation of human triple negative breast cancer cells

**DOI:** 10.37796/2211-8039.1695

**Published:** 2026-03-01

**Authors:** Che-Yi Chao, Woei-Cheang Shyu, Chih-Lung Lin, En-Pei Isabel Chiang, Yueh-Hsiung Kuo, Feng-Yao Tang

**Affiliations:** a Department of Food Nutrition and Health Biotechnology, Asia University, Taichung 41354, Taiwan; b Department of Medical Research, China Medical University Hospital, Taichung 40402, Taiwan; c Graduate Institute of Biomedical Science, China Medical University, Taichung 40604, Taiwan; d Translational Medicine Research Center, China Medical University Hospital, Taichung 40402, Taiwan; e Department of Neurosurgery, Asia University Hospital, Taichung 41354, Taiwan; f Department of Occupational Therapy, Asia University, Taichung 41354, Taiwan; g Department of Food Science and Biotechnology, National Chung Hsing University, Taichung 402, Taiwan; h Innovation and Development Center of Sustainable Agriculture (DCSA), National Chung Hsing University, Taichung 402, Taiwan; i Department of Chinese Pharmaceutical Sciences and Chinese Medicine Resources, China Medical University, Taichung 40604, Taiwan; j Department of Biotechnology, Asia University, Taichung 41354, Taiwan; k Biomedical Science Laboratory, Department of Nutrition, China Medical University, Taichung 40604, Taiwan

**Keywords:** Decyl caffeate, Cell signaling, Cell cycle arrest, Apoptosis, Triplenegative breast cancer

## Abstract

**Background:**

Over recent decades, considerable attention has been directed toward the discovery of novel compounds capable of targeting survival-related signaling networks as therapeutic candidates for triple-negative breast cancer (TNBC). Central to TNBC pathobiology are the Akt/mTOR and MAPK/ERK signaling axes, both contribute to tumor progression and therapeutic resistance. Caffeic acid (CA), a naturally derived phenolic compound with anti-inflammatory activity, has previously been investigated for its anti-cancer potential.

**Purpose:**

In the present study, we explored the therapeutic value of newly synthesized CA derivatives in TNBC models using both cellular and animal based systems.

**Methods:**

The anti-tumor efficacy of these CA derivatives was examined through a series of functional assays, including cell proliferation, clonogenicity, cell cycle profiling, apoptosis quantification, ELISA, western blotting, and histopathological analysis.

**Results:**

Among the tested derivatives, decyl caffeate (DC) demonstrated the most pronounced inhibitory effects on TNBC cell growth, significantly decreasing viability, colony formation, and enhancing cisplatin responsiveness (*P* < 0.05). DC induced G2/M phase arrest in MDA-MB-468 cells, accompanied by suppression of cyclin B1 and CDK1 expression. In addition, DC downregulated both total and phosphorylated c-Myc and reduced secretion of TGF-α, a key ligand for EGFR. Apoptotic responses were evident through upregulation of Bax, cleaved caspase3, and cleaved-PARP. Mechanistic analysis revealed that these effects were mediated via concurrent inactivation of the Akt/mTOR and MAPK/ERK signaling pathways. Oral administration of DC in a murine TNBC xenograft model significantly suppressed tumor growth *in vivo*.

**Conclusion:**

Altogether, these results highlight DC as a promising bioactive compound that targets essential oncogenic pathways in TNBC and support its potential for further preclinical development.

## 1. Introduction

Breast cancer represents the most prevalent malignancy diagnosed in women and remains a primary contributor to cancer-related mortality worldwide [1]. Recent studies have indicated a marked increase in breast cancer incidence among women in Asia, now exceeding rates commonly reported in Western countries [2,3]. This disease is known for its molecular complexity and is categorized into subtypes based on the expression profiles of key receptors, including estrogen receptor (ER), progesterone receptor (PR), and human epidermal growth factor receptor 2 (HER2) [4]. Tumors that lack all three receptors are classified as triple-negative breast cancer (TNBC), a particularly aggressive subtype associated with poor clinical prognosis and limited therapeutic options [5]. A significant challenge in managing TNBC is its resistance to conventional therapies, which often arises from molecular alterations such as gene amplification, activation of compensatory kinase pathways, and remodeling of intracellular signaling networks that collectively enable cancer cells to resist apoptosis and continue proliferating [6,7]. To date, only a few effective targeted treatments are available for TNBC, emphasizing the urgent need for alternative therapeutic strategies. Among molecular targets, receptor tyrosine kinases—especially the epidermal growth factor receptor (EGFR)—have emerged as central drivers in TNBC pathophysiology and are implicated in enhancing tumor cell survival and proliferation [8,9]. EGFR is activated by ligands such as transforming growth factor-alpha (TGF-α), initiating downstream signaling cascades including the PI3K/Akt/mTOR, MAPK/ERK, and c-Myc pathways [10]. This evidence indicates that the nuclear localization and transcriptional activity of c-Myc are regulated via phosphorylation at specific residues, notably serine 62 and threonine 58, enhancing its stability and oncogenic function [11]. Moreover, the PI3K/Akt axis also influences the nuclear translocation of transcriptional regulators like β-catenin, which in turn upregulates genes involved in cell cycle progression, including c-Myc and cyclins. The activation of c-Myc promotes cyclin-dependent kinase (CDK) activity— particularly through cyclin B1 and CDK1—which are critical mediators of the G2/M phase transition in the cell cycle [12]. Persistent activation of PI3K/Akt/mTOR and MAPK/ERK signaling has been closely associated with TNBC progression, treatment resistance, and adverse clinical outcomes [13]. As a result, therapeutic agents capable of simultaneously targeting both pathways are being actively investigated for their potential in treating this challenging breast cancer subtype [14,15].

Naturally derived compounds have attracted considerable attention due to their potential to modulate oncogenic signaling pathways. Phytochemicals from dietary sources, particularly coffee, have shown promise in cancer chemoprevention [16]. Caffeine, a major bioactive compound in coffee, has been widely studied in preclinical models and exhibits inhibitory effects on breast tumor growth [16]. Caffeic acid (CA) has been shown to interfere with ERK1/2 activation in murine models of skin carcinogenesis, thereby reducing tumor development [17]. Structurally related analogs of CA, including caffeic acid phenethyl ester (CAPE) and caffeic acid phenylpropyl ester (CAPPE), have demonstrated significant anticancer properties in both breast and colon cancer models [18–22].

Given the therapeutic potential of compounds targeting multiple oncogenic pathways, this study was conducted to investigate the anticancer activities of novel CA derivative, decyl caffeate (DC), with a specific focus on its ability to concurrently inhibit the Akt/mTOR and MAPK/ERK signaling cascades. We employed both *in vitro* cell-based assays and *in vivo* xenograft models to examine their mechanisms of action and evaluate their efficacy in triple-negative breast cancer.

## 2. Materials and methods

### 2.1. Cell lines, reagents, and materials

**Fig. 1:**
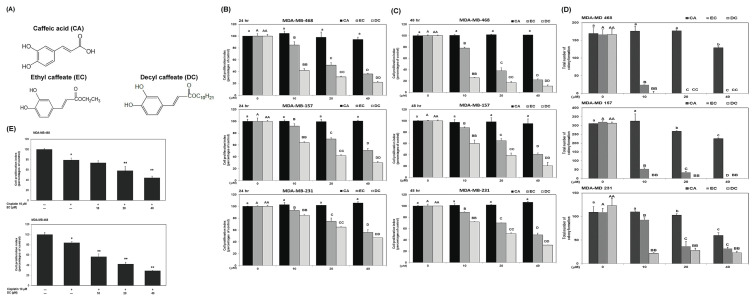
CA derivatives suppress cell proliferation and clonogenic growth in TNBC cell lines. (A) Structural representations of CA, EC, and DC. (B, C) MDA-MB-468, MDAMB-157, and MDA-MB-231 cells were treated with 0, 10, 20, or 40 μM of CA derivatives for 24 h (B) and 48 h (C). Cell viability was assessed using the MTT assay. (D) Long-term proliferative capacity was evaluated through colony formation assays. Distinct lowercase, uppercase, or double uppercase letters denote statistically significant differences (P < 0.05) within the CA, EC, and DC treatment sets, respectively. (E) Synergistic effects of EC or DC (0–40 μM) with 10 μM cisplatin on MDA-MB468 cell viability were measured after 24 h. P < 0.05: single asterisk (*) denotes significance versus the untreated control; double asterisk (**) versus cisplatin alone.

Triple-negative breast cancer (TNBC) cell lines— MDA-MB-468 (ATCC® HTB-132), MDA-MB-157 (ATCC® HTB-24), and MDA-MB-231 (ATCC® HTB26)—were acquired from the American Type Culture Collection (ATCC, Walkersville, MD, USA) and validated for authenticity. MDA-MB-468 cells represent the basal-A TNBC subtype and are characterized by PTEN deletion and EGFR amplification. MDA-MB-231 and MDA-MB-157, both basal-B subtype lines, harbor KRas mutation and PTEN deletion, respectively. Antibodies against phospho-Akt (Thr308 and Ser473), total Akt, phospho-mTOR (Ser2448), total mTOR, phospho-ERK1/2 (Thr202/Tyr204), Bax, cleaved caspase-3, cleaved-PARP, c-Myc, phospho-c-Myc (Ser62), CDK1, and Lamin A were obtained from Cell Signaling Technology (Danvers, MA, USA). Anti-β-actin, caffeic acid (CA), and DMSO were sourced from Sigma-Aldrich (St. Louis, MO, USA). TGF-α ELISA kits were purchased from Thermo Fisher Scientific (Waltham, MA, USA). Plasmid constructs (pBABE-cyclin B1 and empty vector) were acquired from Addgene (Watertown, MA, USA). Protein extraction kits for cytoplasmic and nuclear fractions were supplied by Pierce Biotechnology (Rockford, IL, USA). Propidium iodide and anti-cyclin B1 antibody were purchased from BD Biosciences (Franklin Lakes, NJ, USA). Leibovitz’s L-15 medium and fetal bovine serum (FBS) were provided by Invitrogen (Carlsbad, CA, USA). Synthetic CA derivatives—ethyl caffeate (EC) and DC (Fig. 1A)—were generously provided by Dr. Y. H. Kuo (China Medical University, Taichung, Taiwan).

### 2.2. Cell culture and treatment

TNBC cells were maintained in L-15 medium supplemented with 10 % FBS, 2 mM l-glutamine, and 1.5 g/L sodium bicarbonate. For treatment, CA, EC, and DC were dissolved in DMSO and mixed with culture medium containing 10 % FBS to reach final concentrations of 0, 10, 20, or 40 μM. Control cells were exposed to 0.05 % DMSO to serve as solvent controls. Treatments were applied for 24 h.

### 2.3. Viability assay

The MTT assay was utilized to determine cell viability. TNBC cell lines were seeded into 24-well plates at 2 × 10^4^ cells per well and exposed to CA, EC, or DC at various concentrations (0–40 μM). After 24 or 48 hours (h) of treatment, media were replaced with 0.5 mg/mL MTT reagent and incubated for 2 h. The resulting formazan crystals were solubilized in isopropanol, and absorbance was measured at 570 nm. Each experimental condition was analyzed in triplicate. To evaluate the synergistic effects of cisplatin (10 μM) or the inhibitory action of the pan-caspase inhibitor Z-VAD-FMK (50 μM), TNBC cells were treated with EC or DC, in the presence or absence of these reagents (cisplatin or Z-VAD-FMK) for 24 h. After the treatment period, cell viability was assessed using the MTT assay as previously described.

### 2.4. Colony formation assay

To evaluate long-term proliferation, TNBC cells were seeded into culture dishes and treated with CA, EC, or DC (0–40 μM) for seven days. A small population of treatment-resistant viable cells remained, and their ability to form colonies was assessed as a measure of long-term proliferative capacity under sustained cytotoxic stress. Colonies were fixed and stained with 0.01 % crystal violet. Clusters containing at least 50 cells were counted under a microscope.

### 2.5. TGF-α quantification via Enzyme-Linked Immunosorbent Assay (ELISA)

MDA-MB-468 cells were incubated with CA or DC (0–40 μM) in 24-well plates for 24 h. Cell supernatants were collected and assayed for TGF-α secretion using a commercial ELISA kit according to the supplier’s protocol.

### 2.6. Cell cycle profiling

For analysis of cell cycle distribution, synchronized TNBC cells (1 × 10^5^ per dish) were cultured in 3-cm dishes and treated with CA or DC (0–40 μM) for 24 h. At the end of experiment, floating cells were removed by gentle washing with phosphate buffer saline (PBS). The remaining adherent viable cells were harvested, fixed, and stained with PI. DNA content was analyzed via flow cytometry (FACS Canto, BD Biosciences), and data were interpreted using dedicated software.

### 2.7. Apoptosis detection using Annexin V/PI

To assess apoptotic induction, TNBC cells were treated with CA or DC (0–40 μM). Viable cells were collected after removing dead cells by washing with PBS as described above. After 24 h, 5 × 10^5^ cells were stained with Annexin V-FITC and PI in binding buffer. Following 5 min of dark incubation at room temperature, samples were analyzed using flow cytometry. Apoptotic populations were identified based on fluorescence signal intensity in FITC and PE channels.

### 2.8. Cell transfection

MDA-MB-468 cells at around 80 % confluence were transfected with pBABE-cyclin B1 or an empty pBABE vector using Lipofectamine (Thermo Fisher Scientific). After transfection, stable clones were selected using puromycin for 14 days.

### 2.9. Western blot analysis

Cytoplasmic and nuclear proteins were extracted using fractionation kits with protease/phosphatase inhibitors. Following centrifugation at 12,000 × g for 10 min, supernatants and pellets were collected as cytoplasmic and nuclear fractions, respectively. Protein concentrations were measured and equal amounts (60 μg) were resolved via SDS-PAGE and transferred onto PVDF membranes. Target proteins were detected with specific primary antibodies and visualized by chemiluminescence. Actin and Lamin A were used as internal loading controls for cytoplasmic and nuclear proteins, respectively.

### 2.10. Xenograft model for in vivo tumor growth

To assess tumor growth inhibition *in vivo*, MDA-MB-468 cells (1 × 10^6^ cells in 0.1 mL medium) were orthotopically injected only once into the mammary fat pad of 4-week-old female NOD SCID mice (17–20 g, National Laboratory Animal Center, Taipei, Taiwan). Animals were housed under specific pathogen-free conditions and fed Lab 5010 diet throughout the experiment. Post-inoculation, mice were randomized into control and treatment groups (n = 6 each). The treatment group received DC (2 mg/kg/day) in corn oil via oral gavage (0.15 mL total volume), while controls received corn oil alone. Tumor volume was measured weekly using the formula: volume = 0.524 × L1 × (L2)^2^. Body weight and food intake were monitored to assess general health and exclude dietary confounding.

### 2.11. Histological analysis of tumor and liver tissues

At the study endpoint, tumor and liver tissues were frozen, sectioned (5 μm), fixed with 4 % paraformaldehyde, stained with H&E, and examined for malignancy and hepatic toxicity. Six randomly selected fields per tissue section were imaged at 100× and 200× magnification using an Olympus BX-51 microscope and DP-71 digital camera.

### 2.12. Statistical analysis

All experimental data were processed using SYSTAT software. One-way ANOVA was applied to identify differences among groups, with *P* < 0.05 249 considered statistically significant. Duncan’s multiple range test was used for post 250 hoc comparisons. Student’s *t*-test was performed to assess differences in protein 251 expression between treatment and control groups.

## 3. Results and discussion

### 3.1. CA derivatives suppress cell proliferation and clonogenic growth in TNBC cell lines

To assess the anti-proliferative effects of CA and its derivatives, EC and DC, three TNBC cell lines—MDA-MB-468, MDA-MB-157, and MDA-MB-231—were treated with increasing concentrations (0, 10, 20, 40 μM) for 24 h and 48 h. As shown in Fig. 1B and C, both EC and DC significantly reduced cell viability in a dose-and time-dependent manner. EC treatment decreased MDA-MB-468 cell viability by 15 %, 49 %, and 64 % at 10, 20, and 40 μM after 24 h, with similar reductions observed in MDA-MB-157 and MDA-MB-231 cells. In comparison, DC treatment resulted in greater inhibition, especially in MDA-MB-468 cells, with viability reduced by 58 %, 69 %, and 78 % at the same concentrations. At 48 h, EC (40 μM) reduced MDA-MB-468 viability by up to 78 %, while DC (40 μM) showed enhanced efficacy, inhibiting up to 89 % of cell growth. The IC_50_ values for EC and DC in MDA-MB-468 cells were 20.5 μM and 7.2 μM, respectively, indicating DC’s superior potency. Clonogenic assays confirmed these effects. DC markedly impaired colony-forming ability in all three cell lines (Fig. 1D; *P* < 0.05). Furthermore, both compounds sensitized MDA-MB-468 cells to cisplatin (10 μM), significantly amplifying its cytotoxic effects when co-administered (Fig. 1E). DC exhibited stronger synergy than EC, suggesting it may enhance chemotherapeutic efficacy in resistant TNBC cells. Our results also show that the inhibitory effect of DC alone was indeed substantial and suggests that the tested dose of DC (40 μM) may be approaching its maximum cytotoxic effect. It may have limited the additive or synergistic effect observed when combined with cisplatin (Fig. 1B and E). Our results further showed that DC (at dosage of 40 μM) had no significant impact on other normal cells such as human umbilical vein endothelial cells (HUVECs) (Data not shown). These findings support the conclusion that DC exerts a selective inhibitory effect on breast cancer cells. Our findings reveal that DC, a novel derivative of CA, significantly inhibited the proliferation of human TNBC cells *in vitro*, as demonstrated by proliferation assay and colony formation assay.

### 3.2. DC induces G2/M phase cell cycle arrest via downregulation of cyclin B1, CDK1, and c-Myc

**Fig. 2:**
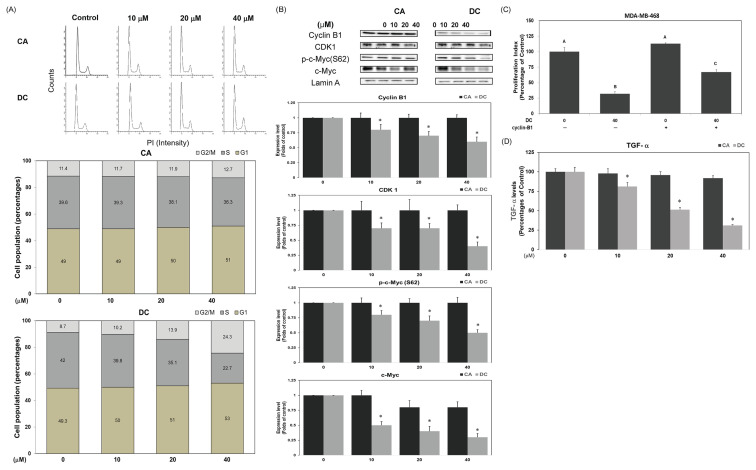
DC induces G2/M Phase Cell Cycle Arrest via Downregulation of Cyclin B1, CDK1, and c-Myc. (A) Cell cycle distribution was determined via flow cytometry after 24-h treatment with CA or DC (0–40 μM) in complete L-15 medium. (B) Expression of cyclin B1, CDK1, phospho-c-Myc (Ser62), and total c-Myc in nuclear extracts was analyzed by Western blotting assay. Lamin A served as the internal control. A single asterisk (*) denotes significance versus the untreated control. (C) Cells transfected with a cyclin B1-expressing plasmid or empty vector were exposed to DC (0 or 40 μM) for 24 h, and viability was assessed via MTT assay. Distinct capital letters denote significant differences (P < 0.05). (D) TGF-α levels in conditioned media were measured after treatment with CA or DC (0–40 μM) for 24 h. A single asterisk (*) indicates P < 0.05 versus the untreated control.

Given the potent anti-proliferative effects, we investigated DC’s impact on cell cycle dynamics in MDA-MB-468 cells. Flow cytometry revealed that DC, but not CA, induced a marked accumulation of cells in G2/M phase, with percentages rising to 10.2 %, 13.9 %, and 24.3 % at 10, 20, and 40 μM, respectively (Fig. 2A). Western blot analysis showed a concentration-dependent decrease in cyclin B1 and CDK1 levels (Fig. 2B). DC also reduced both total and phosphorylated c-Myc (Ser62), a transcription factor that regulates cell cycle progression. To confirm the involvement of cyclin B1, over-expression experiments were performed. MDAMB-468 cells transfected with cyclin B1 plasmid restored proliferation in the presence of DC (Fig. 2C), verifying its functional role in DC-mediated growth arrest. Moreover, DC downregulated TGF-α, an EGFR ligand involved in survival signaling (Fig. 2D), supporting a multi-targeted mechanism. Previous studies have linked cyclin B1 and CDK1 to TNBC proliferation and c-Myc to G2/M transition via regulation of these proteins [23–26]. Our findings align with this, indicating that DC arrests cell cycle progression by disrupting c-Myc/cyclin B1/CDK1 signaling in TNBC cells.

### 3.3. DC promotes apoptosis in TNBC cells via caspase-dependent mechanisms

**Fig. 3:**
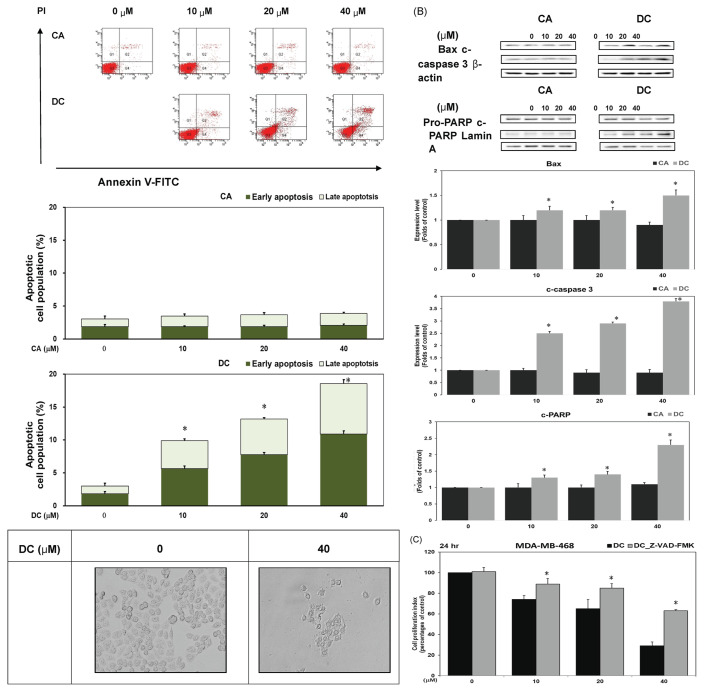
DC promotes apoptosis in TNBC Cells via caspase-dependent mechanisms. (A) Flow cytometry was used to assess apoptosis via annexin V-FITC and PI staining after 24-h exposure to CA or DC. Quantification of apoptotic cells (early and late stages); data represent means ± standard deviation (SD) from three independent replicates. Representative micrographs depicting the morphological alterations in MDA-MB-468 cells following treatment with 40 μM DC were presented. (B) Western blotting was performed on cytoplasmic and nuclear extracts to assess Bax, cleaved caspase-3, cleaved-PARP, and pro-PARP expression. Actin and lamin A served as cytoplasmic and nuclear controls, respectively. A single asterisk (*) denotes significant differences versus control. (C) Cells were treated with DC (0–40 μM) in the presence or absence of pancaspase inhibitor Z-VAD-FMK (50 μM), followed by viability assessment. A single asterisk indicates significance compared to the control subgroup.

To determine whether the anti-proliferative effect of DC involved apoptosis, MDA-MB-468 cells were stained with annexin V-FITC and PI, followed by flow cytometry. DC treatment at 10, 20, and 40 μM dose-dependently increased in early (5.6, 7.8 and 10.9 %) and late (4.3, 5.4, and 7.6 %) apoptotic cell populations (Fig. 3A). Representative images displaying the morphological characteristics of MDA-MB-468 cells after exposure to 40 μM DC were also provided in Fig. 3A. Western blot analysis confirmed apoptosis activation, as indicated by increased levels of Bax and cleaved caspase-3 in the cytoplasm and cleaved-PARP in the nucleus (Fig. 3B). Co-treatment with the caspase inhibitor ZVAD-FMK significantly restored cell viability in DC-treated cells (Fig. 3C), demonstrating that DC-induced apoptosis is caspase-mediated. These results confirm that DC promotes programmed cell death in TNBC cells by activating the intrinsic apoptotic pathway and support its role as a potential pro-apoptotic agent in breast cancer therapy.

### 3.4. DC disrupts Akt/mTOR and MAPK/ERK pathways in TNBC cells

**Fig. 4:**
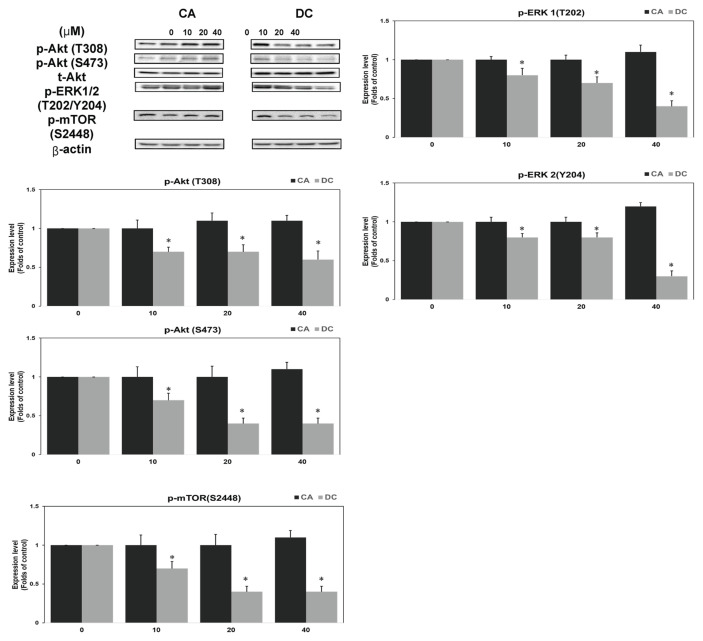
DC Disrupts Akt/mTOR and MAPK/ERK Pathways in TNBC Cells. Cells were treated with DC (0–40 μM) for 24 h in FBS-supplemented L-15 medium. Western blotting assay of phospho-Akt (Thr308 and Ser473), phospho-ERK1/2 (Thr202/Tyr204), and phospho-mTOR (Ser2448) was performed. Total levels of Akt and β-actin were included as controls. A single asterisk (*) denotes statistically significant differences from untreated controls (P < 0.05).

Since TNBC is frequently driven by overactive EGFR signaling, we next examined whether DC interferes with its downstream pathways. Results demonstrated that DC treatment significantly reduced the phosphorylation of Akt and mTOR, two critical components of the PI3K/Akt/mTOR axis. Additionally, DC decreased the phosphorylation of ERK1/2, indicating inhibition of the MAPK/ERK pathway (Fig. 4). These dual inhibitory effects provide a mechanistic basis for DC’s anti-tumor activity. Previous studies have shown that reduced Akt activation leads to decreased mTOR phosphorylation, while mTOR inhibition downregulates c-Myc expression [27,28]. Cyclin B1, regulated by both Akt/mTOR and MAPK/ERK signaling, is also suppressed under dual pathway inhibition [29]. Recent evidence further supports that combined targeting of PI3K/Akt/mTOR and MAPK/ERK pathways enhances therapeutic efficacy in TNBC [30]. Our findings confirm that DC inhibits key survival signaling cascades, contributing to both cell cycle arrest and apoptosis. This dual-target approach may overcome resistance mechanisms and improve treatment outcomes in TNBC.

### 3.5. DC suppresses tumor growth in a TNBC xenograft model

**Fig. 5:**
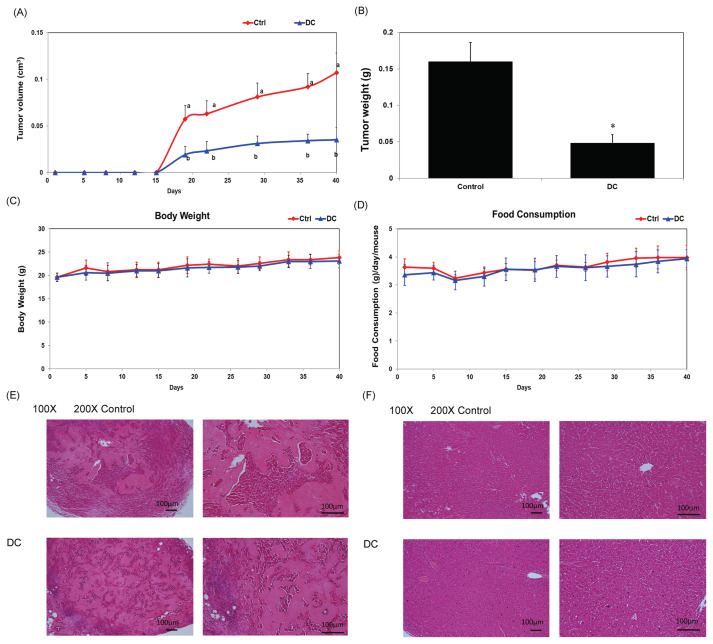
DC Suppresses Tumor Growth in a TNBC Xenograft Model. NOD SCID mice bearing MDA-MB-468 xenografts were treated with DC (2 mg/kg/day) or vehicle for six weeks. (A) Tumor volume was recorded weekly. Distinct letters at each time point indicate significant differences (P < 0.05). (B) Tumor weights at study endpoint; a single asterisk (*) indicates P < 0.05. Body weight (C) and food consumption (D) were monitored following DC administration in xenograft-bearing mice. Data are expressed as mean ± SD, and no statistically significant differences were observed between treatment and control groups. Histological examination of tumor (E) and liver tissues (F) (H&E stained) at 100× and 200× magnification. Blue and red indicate hematoxylin-stained nuclei and eosin-stained cytoplasm, respectively.

To evaluate the *in vivo* efficacy of DC, a xenograft model was established using MDA-MB-468 cells implanted into NOD SCID mice. Oral administration of DC (2 mg/kg/day) for six weeks resulted in significantly reduced tumor volume and weight compared to controls (Fig. 5A and B; *P* < 0.05). Changes in body weight (Fig. 5C) and dietary intake (Fig. 5D) were measured following DC administration in xenograft-bearing mice. No statistically significant differences were observed between DC-treated and control groups. Histological analysis of tumor sections confirmed the inhibition of tumor progression in the DC-treated group (Fig. 5E). No evidence of liver toxicity was observed (Fig. 5F). These *in vivo* data are consistent with *in vitro* findings and strongly support the therapeutic potential of DC in the treatment of triple-negative breast cancer.

## 4. Conclusions

**Fig. 6:**
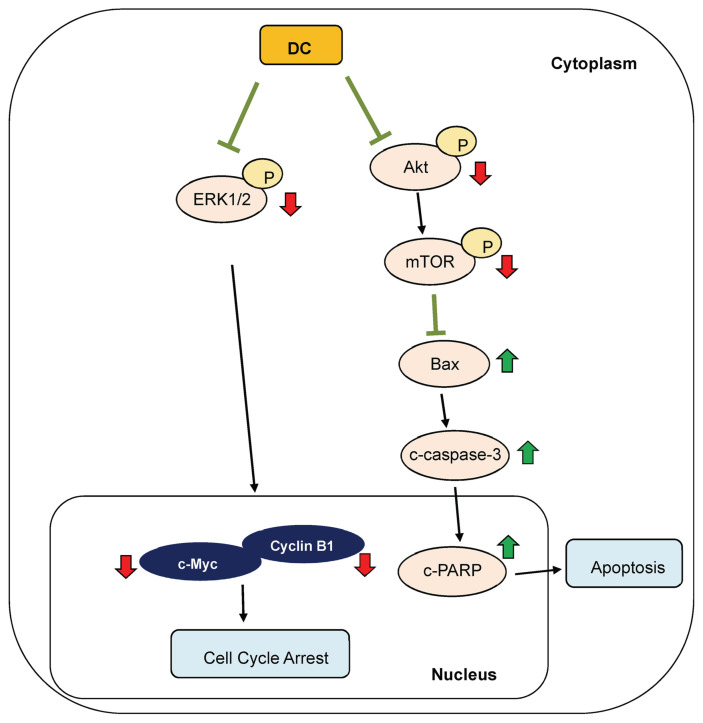
Schematic overview of DC-mediated inhibition of PI3K/Akt/mTOR and MAPK/ERK signaling in TNBC cells. The proposed mechanism illustrates how DC interferes with critical survival and cell cycle pathways, leading to growth inhibition and apoptosis in TNBC. : induction; : suppression.

This study demonstrates that DC, a synthetic derivative of caffeic acid, exerts potent anti-tumor effects in triple-negative breast cancer models. DC significantly inhibits cell proliferation, induces G2/M phase arrest, and activates caspase-dependent apoptosis *in vitro*. Mechanistically, these effects are mediated by the downregulation of cyclin B1, CDK1, c-Myc, and TGF-α, along with suppression of the Akt/mTOR and MAPK/ERK signaling pathways. *In vivo*, DC reduced tumor growth without detectable toxicity. As illustrated in Fig. 6, DC disrupts multiple oncogenic pathways involved in TNBC progression. These results highlight DC as a promising inhibitor with therapeutic potential for managing aggressive breast cancer subtypes lacking targeted treatments.
